# From Pixels to Patterns: Trait Plasticity and Species Overlap of *Calanus* spp. in Arctic Fjords

**DOI:** 10.1002/ece3.71366

**Published:** 2025-05-22

**Authors:** Emilia Trudnowska, Ali Bukhari, Marta Gluchowska, Mads Schultz, Irina Smolina, Joanna Stoń‐Egiert, Jędrzej Świeżewski, Kaja Balazy

**Affiliations:** ^1^ Institute of Oceanology Polish Academy of Sciences Sopot Poland; ^2^ Appsilon Data for Good Warszawa Poland; ^3^ Nord University Bodø Norway

**Keywords:** Arctic, artificial intelligence, Atlantification, *Calanus*, intraspecific variability, traits

## Abstract

The marine ecosystems of the Svalbard archipelago are now propelled towards a new climatic state owing to the ongoing “Atlantification” process. Fjords represent valuable natural laboratories hosting both local and advected populations of zooplankton. Our goal was to study life‐history traits of two *Calanus* species ( *C. glacialis* and *C. finmarchicus* ) across slightly different environments in order to better understand their functioning within Arctic ecosystems undergoing substantial transformations. We hypothesized that life‐history traits of *Calanus* copepods (CV life stage) such as size, pigmentation, lipid content, diet, parasite presence, and stage structure would differ across four hydrographically distinct fjords (Hornsund, Isfjorden, Kongsfjorden, van Mijenfjorden) of Spitsbergen. Morphological size‐based species identification via stereomicroscopy was supported by molecular methods. Manual image‐based measurements of body size and lipid sack area were augmented by machine learning image analyses. Visual color intensity estimations were assisted by HPLC quantification of astaxanthin concentrations. Trophic variability was assessed via stable isotope analyses. The observed substantial variability in the life‐history traits highlights their high plasticity and suggests that the traditional morphological distinctions between two *Calanus* species are becoming increasingly ambiguous. This underscores the need to incorporate genetic tools in ecological studies. The observed variability likely results from the coexistence of several cohorts of both species, including a mixture of local and advected populations. Moreover, each generation could be characterized by different traits depending on their source location and recruitment timing. These findings imply that under progressing “Atlantification” multiple adaptive responses may be expected, including reduced body size, accelerated development, mixed reproductive strategies, decreased pigmentation, shifts in diet, diversified lipid storage strategies, and increased parasite prevalence. Additionally, we introduce a machine learning‐based tool for automatic assessment of key traits, such as body size and lipid content from images.

## Graphical abstract

The reported here high variability in traits of Calanus reveals their high potential for adaptability and/or some resilience to the environmental changes. Due to climate‐induced changes, Calanus copepods will likely further experience: (1) size reduction, (2) faster development, (3) increased competition, both between species and among generations, (4) mixed reproduction strategies, (5) modified pigmentation camouflage, (6) diet flexibility in terms of composition and timing, (7) diverse lipid accumulation strategies affecting their contribution to lipid pump, and (8) the presence of parasites.

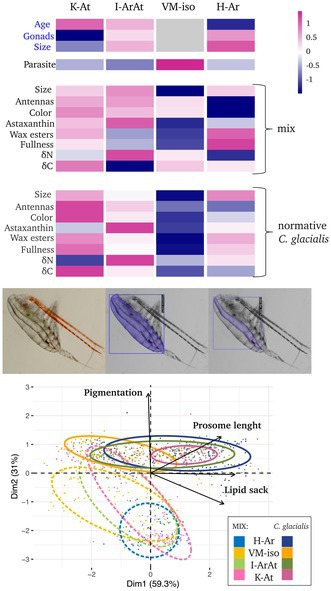

## 1 Introduction

In light of global climate‐induced changes, there is an urgent need for appropriate observational metrics that can be used as a reliable background for understanding the nature and worldwide consequences of those changes. However, before we can start thinking globally, we need to find basic indicators that may be useful in tracking changes at the local scale. Some groups of animals have especially highly recognized characteristics that are good indicators of environmental changes. In the marine ecosystems of the northern hemisphere, *Calanus* copepods, which are key components of zooplankton biomass, often serve this role. The model role of specific species is owing to their various life‐history strategies and various distribution patterns (Gabrielsen et al. 2012; Choquet et al. 2017; Møller and Nielsen 2019; Strand et al. 2020). *Calanus* copepods also have important ecological and commercial value (Beaugrand 2005; Pedersen et al. 2014), both of which are attributable to their high lipid content and crucial function in the transfer of matter and energy between primary producers and higher trophic levels (Falk‐Petersen et al. 2009; Renaud et al. 2018). They are also valued as important vectors of “the lipid pump,” by which the lipid rich individuals that perform seasonal ontogenic migrations to survive periods of darkness transport organic matter towards depth (Jónasdóttir et al. 2015; Tarling, Belcher, et al. 2022). Due to increasing plans for commercial harvesting of *Calanus* as a source of lipids and antioxidants, both for human consumption and aquaculture industry (Pedersen et al. 2014; Espinasse et al. 2018), the development of conservation strategies will soon become a necessity.

As a rule, *C. glacialis* should be larger in size than *C. finmarchicus* and have red antennas (Nielsen et al. 2014; Choquet et al. 2018; Lindeque et al. 2022). Individuals conforming to these criteria are here defined as the normative *C. glacialis* . However, owing to recent molecular species identifications, it is known that those morphological attributes are not sufficient for reliable species identification. For example, crucial traits such as size and lipid content can be similar for two sibling species, Atlantic origin *C. finmarchicus* and local Arctic *C. glacialis* , making them equally attractive for predators (Renaud et al. 2018). However, for some reason, little auks are extremely selective for *C. glacialis* even when exposed to Atlantification (Balazy et al. 2023). Additionally, pigmentation as a trait defining their fitness and underwater visibility can also vary greatly (Vilgrain et al. 2023). The plasticity of these traits in *Calanus* was shown to be substantial when distinct regimes were compared: their original water masses and suboptimal conditions (Trudnowska, Balazy, et al. 2020). Because climate‐induced changes directly impact local temperature and food dynamics (Kjellerup et al. 2012; Mackas et al. 2012; Kvile et al. 2014; Friedland et al. 2016; Espinasse et al. 2018; Skjoldal et al. 2021), which are crucial factors for the phenology of *Calanus*, stage structure, and diet are other important traits to be monitored.

In the 21st century, the use of a trait‐based approach became widespread, including ecological studies of zooplankton (Barnett et al. 2007; Litchman et al. 2013, 2021; Hébert et al. 2017; Benedetti et al. 2019). This approach has been recognized to have high potential as a way to provide robust and ecologically meaningful metrics for general observations for cross‐system comparisons (Schneider et al. 2019; Ohman and Browman 2019; Zakharova et al. 2019; Martini 2020; Violle et al. 2007; Loreau 2010). Because in numerous studies, individual‐level traits are mixed with those at the population‐level and are referred to the environment (Dawson et al. 2021), the term trait has frequently drifted from the classic definition (Violle et al. 2007). The new concepts of traits are arising in the view that the trait‐based approaches are at least as diverse as trait ecologists (Dawson et al. 2021).

For trait‐based studies, images of *Calanus* individuals may provide a wealth of information, such as size, pigmentation, lipid content, or the presence of parasites. Typically, it takes a tremendous amount of time and human resources to manually compute those features across hundreds or thousands of photos collected within the scope of field campaigns and research projects. Therefore, a machine learning‐based solution that can segment body parts, such as prosome and lipid sacs, with sufficient accuracy can easily solve this problem by automating the computation process and providing unified and repeatable results (Irisson et al. 2022; Orenstein et al. 2022; Maps et al. 2024).

Previously found clear gradient of life‐history traits of *Calanus* across different oceanic domains of one fjord (Hornsund) (Trudnowska, Balazy, et al. 2020), triggered the next question: Do those traits also vary across neighboring fjords that differ in their hydrographical regimes? For such considerations, the fjords of the Svalbard Archipelago provide a perfect scene, as they are propelled toward a new climatic state due to the “Atlantification” process, but at various rates (Polyakov et al. 2020; Skogseth et al. 2020; Bloshkina et al. 2021; Ingvaldsen et al. 2021). Therefore, these fjords can be treated as natural laboratories that provide similar habitats but that differ in their susceptibility to Atlantic water advection and thus in the extent of the impact of Atlantic‐associated fauna inflow (Basedow et al. 2004; Gluchowska et al. 2016; Trudnowska, Stemmann, et al. 2020).

The coexistence of *C. glacialis* and *C. finmarchicus* species is common in Arctic coastal ecosystems (e.g., Strand et al. 2020; Trudnowska, Stemmann, et al. 2020) but still not completely understood. The relative contribution of both *Calanus* species can be highly variable in advective fjords; for example, in Isfjorden, the dominance of *C. glacialis* (Hatlebakk et al. 2022), the dominance of *C. finmarchicus* (Gluchowska et al. 2016), or high spatial and interannual variability in their relative roles (Szeligowska et al. 2020) have been recorded. Although both species occupy similar ecological niches, their different timing in reproduction reduces competition (Hatlebakk et al. 2022), and they may adapt to climate‐induced changes differently. Our goal was to study life‐history traits of *Calanus* across slightly different environments in order to better understand their functioning within Arctic ecosystems undergoing substantial transformations. We hypothesized that the key life‐history traits of *Calanus* species: body size, pigmentation, lipid content, diet, the presence of parasites, and stage structure would vary across four fjords characterized by different hydrographical regimes. To investigate the extent of trait variability in the CV life stage, we applied a range of complementary and up‐to‐date methodological approaches: morphological size‐based species identification via stereomicroscopy was supported by molecular tools; manual image‐based measurements of body size and lipid sack area were assisted by machine learning analysis; pigmentation was assessed visually from images and through HPLC quantification of astaxanthin concentrations; and trophic variability was assessed via stable isotope composition analysis.

## 2 Materials and Methods

### 2.1 Sampling

**FIGURE 1:**
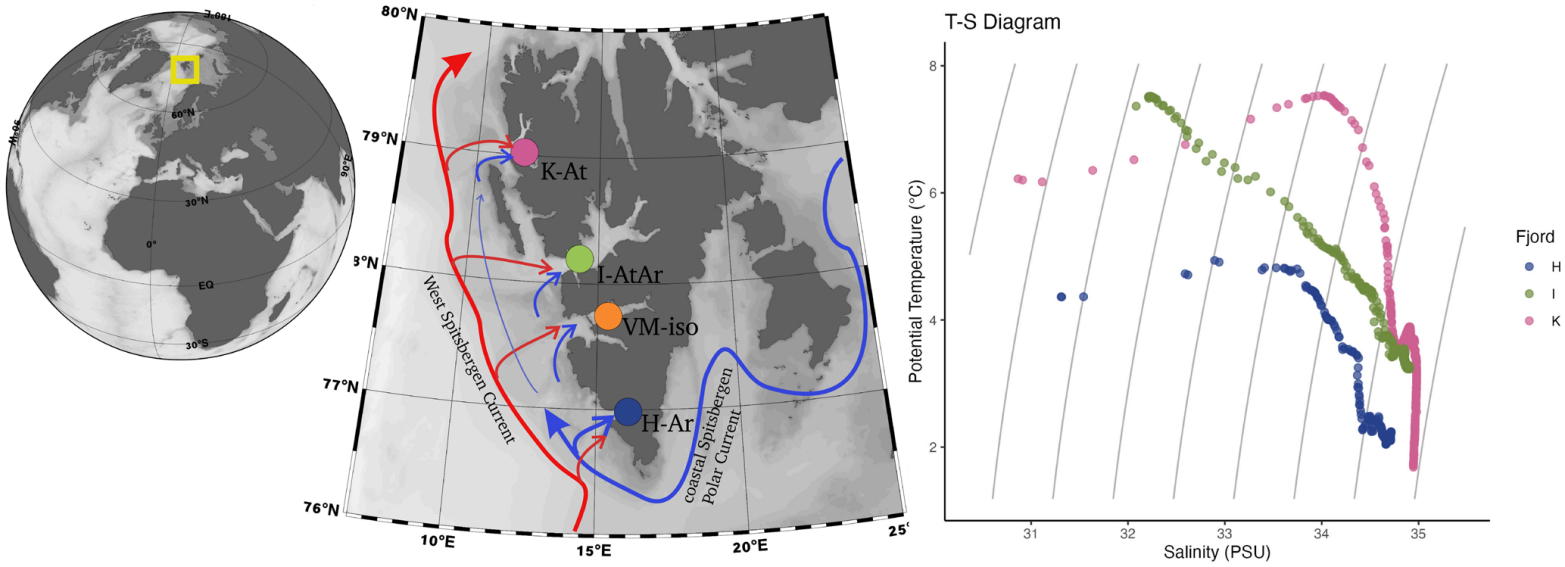
Study area. The Svalbard archipelago is marked by a yellow frame on the global map. The close‐up map presents the pattern of ocean currents along the west Spitsbergen coast, with red arrows representing the Atlantic origin West Spitsbergen Current and blue arrows representing the Arctic origin coastal Spitsbergen Polar Current; the studied fjords are marked by dots of the various colors used throughout the article, with Arctic‐type Hornsund (H‐Ar), isolated Van Mijenfjorden (VM‐iso), variable Isfjorden (I‐AtAr), and Atlantified Kongsfjorden (K‐At); and temperature–salinity (T‐S) diagram from the measurements taken at the time of sampling at the study locations.

The study was performed in the summer of 2019 onboard RV Oceania in 4 fjords of the west Spitsbergen: Van Mijenfjorden (29th of July), Hornsund (2nd August), Isfjorden (5th August) and Kongsfjorden (9th August). These fjords differ in hydrography (Figure 1) because of the different interactions between local and advected water masses (Cottier et al. 2005; Promińska et al. 2017; Strzelewicz et al. 2022). Hornsund, which is situated on the southwestern tip of Spitsbergen, is the fjord most influenced by the cold and fresh coastal Spitsbergen Polar Current and thus has the most Arctic‐type characteristics (Strzelewicz et al. 2022). The lack of a distinct sill at the entrances of Kongsfjorden and Isfjorden leads to easy exchange of the waters, mostly in the form of advection of Atlantic waters (Svendsen et al. 2002; Nilsen et al. 2008; Wiencke and Hop 2016). Consequently, hydrographical conditions in the main basin of Isfjorden are in a state of dynamic exchange between Arctic and Atlantic water masses (Cottier et al. 2005, 2010; Nilsen et al. 2008; Skogseth et al. 2020). Although Kongsfjorden has the most northern location, it is typically considered the most boreal fjord, as it receives twice as much Atlantic water as Hornsund (Svendsen et al. 2002; Promińska et al. 2017; Hop and Wiencke 2019). Van Mijenfjorden differs from the other Spitsbergen fjords in that its mouth is nearly closed by the island Akseløya, restricting water exchange between the fjord basin and coastal water masses. Although the island and sill restrict water exchange between the fjord and coastal waters, basin water is presumed to be exchanged each winter by convection and inflow of coastal water, and bottom waters remain oxygenated, which results in stable conditions for benthic communities (Renaud et al. 2007). Such a classification of fjords has also been applied in other similar studies (Gluchowska et al. 2016, Ormańczyk et al. 2017, Balazy et al. 2019, 2023). To simplify the reading and interpretation of results, the abbreviations indicating each fjord and its characteristics were used throughout the article: Arctic‐type Hornsund (H‐Ar), isolated Van Mijenfjorden (VM‐iso), variable Isfjorden (I‐AtAr), and Atlantified Kongsfjorden (K‐At).

At each study location, two vertical tows of a WP2 net (180 μm mesh size), preceded by a CTD profile, were performed through the water column from the surface to a few meters (~5) above the sea bottom (220 m in Hornsund, 215 in Isfjorden, 300 m in Kongsfjorden). The material collected from the first haul of the net was immediately fixed in a formaldehyde–borax solution as a sample for analysis via stereomicroscopy of *Calanus* abundance, developmental stage composition, gonad maturation stages, and size distribution (Table S1). The material collected by the second net haul was used to select individuals only in the fifth copepodite stage of *Calanus* (CV), which were photographed alive as soon as possible after collection using a digital camera (Olympus SC50 CMOS Color Camera) mounted on an Olympus SZX16 stereomicroscope. The material was stored in a bucket in a refrigerator, and individuals were selected by subsampling to the counting chamber to guarantee their good condition for further analyses. Similarly, as in a previous study (Trudnowska, Balazy, et al. 2020), for quick categorization onboard we assumed that small and large size modes would successfully represent two species ( *C. glacialis* —large, *C. finmarchicus* —small). Each individual was placed in a drop of water prior to making the high‐resolution image. The 922 images were used for the determination and measurements of prosome length, the area of the lipid sac, and pigmentation (Table S1). After taking images, the individuals were preserved for further analyses in alcohol for genetic analysis, pooled by 10 individuals per sample, and frozen at −80°C for stable isotope and HPLC analyses.

Only genetic, astaxanthin, stable isotope, and photo‐based data were available for Van Mijenfjorden, whereas CTD profile and samples fixed in formaldehyde were not collected because of technical issues.

### 2.2 Molecular Identification of Species

Genetic identification as a method to ground truth the assignment of individuals to species/group category was performed for 216 *Calanus* CV individuals (Table S1). The analysis was based on six nuclear insertion–deletion (InDels) markers via a multiplex polymerase chain reaction (PCR) processing by following the protocol of Choquet et al. (2017). Unfortunately, owing to their high plasticity in size, in numerous instances it was not possible to get clear species‐specific samples, as revealed by later genetic identifications. In brief, we found more *C. glacialis* than expected—143 individuals were identified as *C. glacialis* and only 74 as *C. finmarchicus* , while sampling aimed to collect equal numbers of species per site. When only large individuals with red antennas were selected, a 100% agreement between morphological and genetic identification was obtained for K‐At, I‐AtAr, and H‐Ar, and 93% agreement was obtained for VM‐iso (Table 1). However, in H‐Ar and I‐AtAr, it was difficult to obtain distinct *C. finmarchicus* subsamples, where this size mode was also represented by *C. glacialis* . Consequently, we were not able to morphologically distinguish small *C. glacialis* from *C. finmarchicus* , as genetically confirmed *C. glacialis* made 79% (Isfjorden), 56% (Hornsund), 24% (Van Mijenfjorden), and 20% (Kongsfjorden) of smaller sized fractions assumed to be *C. finmarchicus* . As a consequence, throughout the manuscript, a distinction of results into two groups will be used: (i) normative *C. glacialis* (large and pigmented individuals, as the trait of pigmentation has been shown to be reliable on the basis of genetically identified individuals (Figure S1)) and (ii) a mixture of small *C. glacialis* and *C. finmarchicus* .

**TABLE 1:** Genetic verification of *Calanus* copepodites at the fifth developmental stage (CV) across four Svalbard fjords: Arctic‐type Hornsund (H‐Ar), isolated Van Mijenfjorden (VM‐iso), variable Isfjorden (I‐AtAr), and Atlantified Kongsfjorden (K‐At).

Fjord	*C. finmarchicus*	*C. glacialis*	Type	*n*
K‐At		100%	Normative	58
K‐At	80%	20%	Mix	
I‐AtAr		100%	Normative	57
I‐AtAr	21%	79%	Mix	
H‐Ar		100%	Normative	48
H‐Ar	44%	56%	Mix	
VM‐iso		93%	Normative	54
VM‐iso	76%	24%	Mix	

*Note:* Values indicate the percentage of correctly assigned individuals per sample. Samples with > 90% *C. glacialis* assignments were classified as the normative *C. glacialis* category; samples with lower assignment accuracy were designated as the “mix” category, comprising *C. finmarchicus* and/or small *C. glacialis* individuals.

### 2.3 Analyses of Zooplankton Samples

The analyses of zooplankton samples for morphological species identification and abundance estimation were performed via a standard procedure with subsampling (three per sample). For the life stage structure analysis, 890 individuals of *Calanus* were examined via these subsampling procedures (Table S1). The relative abundances of each of the *Calanus* copepodite stages were used to describe the population stage structure. The abbreviations (CI‐CV) refer to five successive copepodite stages of *Calanus* and AF refers to adult females. The reference to typical size discrimination of particular developmental stages into species was based on the assessment performed for the populations from the Hornsund and Kongsfjorden (Kwasniewski et al. 2003; Weydmann and Kwasniewski 2008). The stages in the gonad maturation stages of *Calanus* females included Gs1 and Gs2 as immature stages, Gs3 in the transition to maturity stage, and Gs4 the final maturation stage (Niehoff and Runge 2003; Niehoff 2007).

### 2.4 HPLC

To access the pigmentation, the red carotenoid astaxanthin was detected and quantified via a chromatographic technique (HPLC), which allows the pigments to be separated from a previously prepared mixture, identified on the basis of their absorption properties, and quantified. The pooled samples of 10 *Calanus* CV individuals (Table S1) were lyophilized and weighed before pigment extraction from their cells was conducted. The extraction procedure was based on mechanical grinding in the presence of 90% acetone and sonication (2 min, 20 kHz, Cole Parmer, 4710 Series) for 2 h in the dark. The extract was subsequently clarified and injected onto a C18 LichroCART LiChrospher 100 RP18e analytical column (250 × 4 mm dimensions, 5 μm particle size and 100 Å pore size, Merck). The qualification of astaxanthin was based on the retention time and similarity of the absorbance spectrum to the standard and its quantification on the basis of the response factor obtained during the calibration procedure (Stoń‐Egiert and Kosakowska 2005).

### 2.5 Stable Isotopes

To verify whether diets of two categories differed, the samples of pooled 10 individuals of *Calanus* CV for stable isotope analysis were analyzed via an elemental analyzer (Flash EA 1112, Thermo Scientific, Milan, Italy) coupled with an isotope ratio mass spectrometer (Delta V Advantage with a ConFLo IV interface, Thermo Scientific, Bremen, Germany) according to standard protocols (Lebreton et al. 2012). The results are expressed in the δ unit notation as deviations from standards (Vienna Pee Dee Belemnite for δ13C and N2 in air for δ15N) following the formula: δ13C or δ15N = [(Rsample/Rstandard) − 1] × 103, where R is 13C/12C or 15 N/14 N, respectively. Calibration was performed via reference materials (USGS‐24, IAEA‐CH6, IAEA‐600, USGS‐61, and USGS‐62 for carbon; IAEA‐N2, IAEA‐NO‐3, IAEA‐ 600, USGS‐61, and USGS‐62 for nitrogen). Analytical precision based on the analyses of acetanilide (Thermo Scientific) used as a laboratory internal standard was \0.1 and \0.15‰ for carbon and nitrogen, respectively.

### 2.6 Image‐Based Manual Analysis

The prosome length values of 922 photographed *Calanus* CV copepodites were measured from the tip of the prosome to the distal lateral end of the last thoracic somite (Table S1). The measurements were performed with ImageJ/Fiji free software for image analysis.

The examination of photos also made it possible to distinguish individuals whose internal dark structures resembled Blastodinium (Dinoflagellata) parasites, similar to those described previously (Fields et al. 2015; Cleary et al. 2022).

The visual assessments of the red pigmentation in *Calanus* CV individuals from images were performed according to a color‐coding scheme, with four levels of intensity: 3 (> 50% red), 2 (10%–50%), 1 (< 10%), and 0 (0%, no pigmentation). This was done for different body parts separately: antennae, prosome, legs, and urosome (Trudnowska, Balazy, et al. 2020).

The lipid sacs were manually measured by contouring the sac area by hand on photos of the *Calanus* CV. Fullness by lipids was calculated as a percentage of the lipid sac area within the total area of the prosome, whereas the amounts of wax esters were estimated by applying equations for image‐based Arctic *Calanus* assessments (Vogedes et al. 2010).

### 2.7 Machine Learning to Automate Image‐Based Analyses

The dataset employed for machine learning analyses consisted of 321 high‐resolution (2560 × 1920) images of *Calanus* CV, identical to those used in the manual image analysis. These images are rich in detail, clearly show visible prosomes and lipid sacs, making them ideal for automated image segmentation, which included delineating the prosome and lipid sac from the surrounding environment. The dataset was divided into two random groups: 150 images used for training the model (manually labeled) and 171 images used for testing. While this split leaves more data for testing than usual (a typical split is 4:1), it was dictated by, on the one hand, good training behavior already on the small dataset, and on the other hand, to allow for a more robust evaluation.

The model architecture used was Mask R‐CNN (He et al. 2017), a variant of Convolutional Neural Networks integrated with Region Proposal Networks, to segment and delineate specific body parts such as the prosome and lipid sac. The choice of architecture was dictated by its ability to leverage spatial hierarchies inherent in image data, generating candidate object regions that are then segmented at the pixel level. To increase the resolution and precision of these segmentations, especially around intricate boundaries, we integrated the PointRend technique (Kirillov et al. 2020).

MaskRCNN and PointRend components within each instance were concurrently trained. Since the lipid sac is a spatial subset of the prosome, resulting in double‐labeled pixels, we trained two distinct Mask R‐CNN instances—one for the lipid sac and another for the prosome—both sharing the same architectural parameters. This resulted in two sets of models: a foundational pair (referred to as Model 1 hereafter) and another enhanced with PointRend refinements (referred to as Model 2). The models were trained by leveraging the detectron2 framework (Vuang Pham et al. 2020: https://github.com/facebookresearch/detectron2) for 2000 cycles each, facilitated by two workers and processed in two images per batch, culminating in a training time of approximately 15 min on a Nvidia Tesla K80 GPU per training. To assess the usefulness of the above model and improve upon Model 2 s for calculations of derived metrics, the lipid sac to prosome ratio (LP ratio) was calculated by dividing the segmented lipid sac pixel count by the prosome pixel count. The ratios were computed for both the machine learning models (Model 1 and Model 2) and each of the images from the test set. Independently, an LP ratio was obtained for each of the images via the traditional manual fitting of ovals to match the structures of interest.

Two metrics were used to evaluate the performance of the segmentation models: Intersection over Union (IoU) and the F1‐score. The IoU, defined as the ratio of the intersection of the predicted and ground‐truth segments, and the F1‐score, defined as the harmonic mean of precision.

The tool will be available to any researcher on demand from Appsilon (https://appsilon.com/copepod‐prosome‐and‐lipid‐sac‐segmentation‐with‐machine‐learning/).

### 2.8 Scaling Traits Together

Heat map visualization was used to combine all the studied traits into a comprehensive overview over the fjords and categories. Moreover, morphological traits were scaled together on Principal Component Analysis (PCA). Prior to the analysis, the data were normalized, and the Euclidean distance values were calculated. Analyses were performed in R in libraries: “ggplot2,” “oce,” “FactoMineR,” “factoextra,” “DescTools,” “pheatmap,” and functions: kruskal.test, pairwise.wilcox.test, cor.test, coef, var., fisher.test, PCA, GTest, scale, pheatmap.

## 3 Results

### 3.1 Size Variability

**FIGURE 2:**
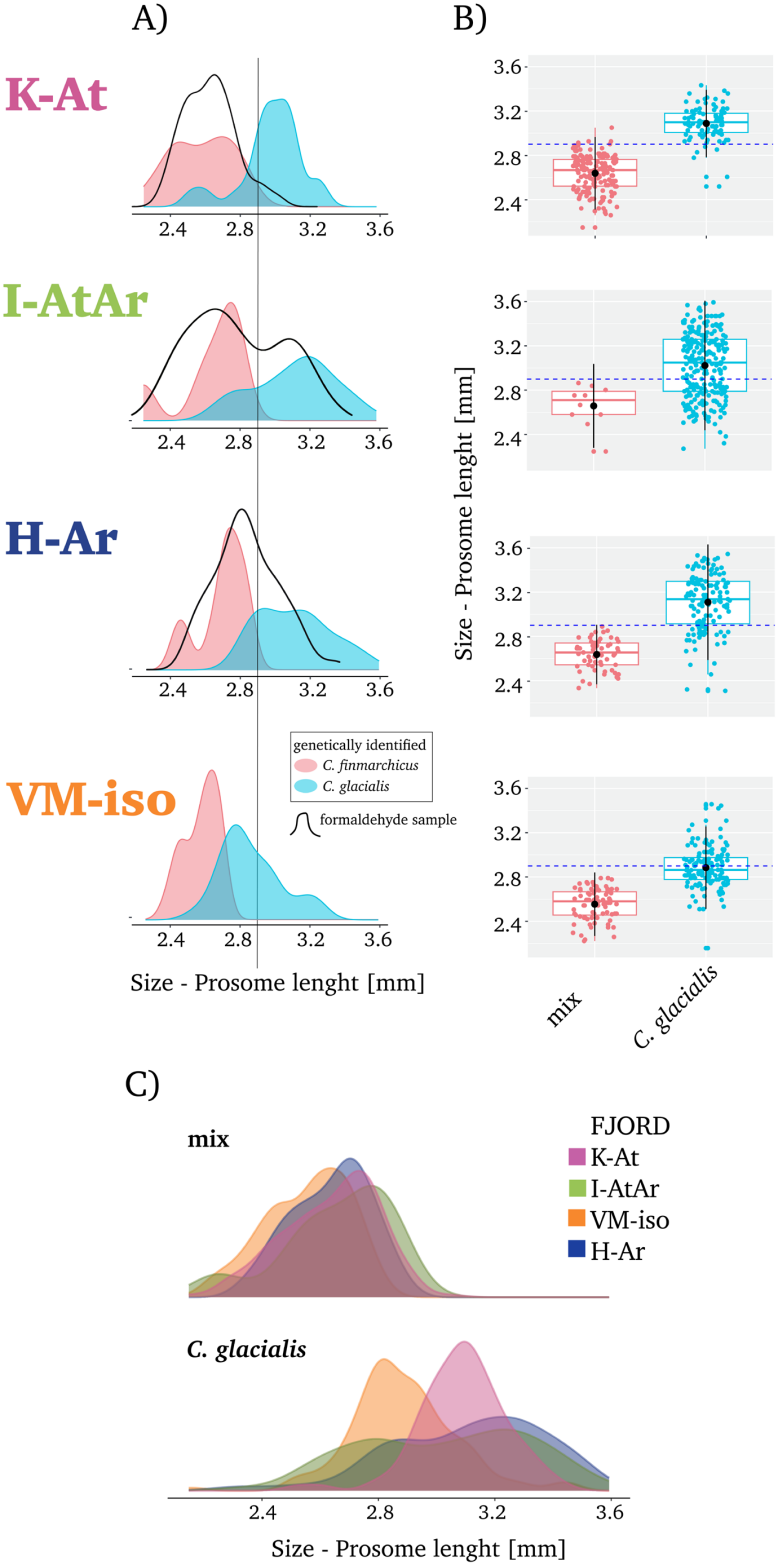
Size variability in the prosome length of the fifth life stage (CV) of *Calanus* in four investigated regions (K‐At: Kongsfjorden; I‐AtAr: Isfjorden; H‐Ar: Hornsund; VM‐iso: Van Mijenfjorden). (A) Kernel density plots of the size distribution of genetically identified individuals (in color) and formaldehyde‐preserved samples (black contour). Solid vertical line indicates literature‐based size limit. Prosome length of all photographed *Calanus* CV individuals: (B) Box plots presenting mean, median, and Q1–Q3 quartiles; dashed horizontal line indicates literature‐based size limit, (C) Kernel density across species categories and fjords.

According to the genetically identified CV individuals, each species reflected two size modes, with slight differences in their size distributions among fjords (Figure 2A). The largest individuals of *C. glacialis* were found in I‐ArAt and H‐Ar (on average 3.11 and 3.09 mm of prosome length, respectively) and the smallest in VM‐iso (2.85 mm). Numerous genetically identified individuals (40) of *C. glacialis* were smaller than the size discrimination limit (2.9 mm) from the literature (Kwasniewski et al. 2003; Weydmann and Kwasniewski 2008), which was especially pronounced in VM‐iso but occurred in each investigated fjord (Figure 2A).

In the formaldehyde samples, individuals of *Calanus* CV from K‐At were generally rather small, with a tendency to have two size modes (2.4 and 2.6 mm) and a slight tail of a larger size fraction (> 2.9 mm) (Figure 2A). In the formaldehyde sample from I‐AtAr, two clearly dominant size modes of *Calanus* CV were observed, with average mean sizes of 2.6 mm and 3.1 mm. In H‐Ar, only one dominating size mode (mean 2.8 mm) was observed when considering the total community of *Calanus* (Figure 2A).

The analysis of all the photographed live individuals also showed that the *C. glacialis* CV can be much smaller than the size limit of 2.9 mm, especially in I‐AtAr (Figure 2B). Even though the size range of the mix category very rarely exceeded the size limit of 2.9, some subtle differences in the size peaks were observed among fjords (Figure 2C). The variability in the size of normative *C. glacialis* was the greatest in I‐AtAr (variance = 0.09) and the lowest in K‐At (variance = 0.02) (Figure 2C).

### 3.2 Life Stage Structure, Gonad Maturation Stages and Presence of Parasites

**FIGURE 3:**
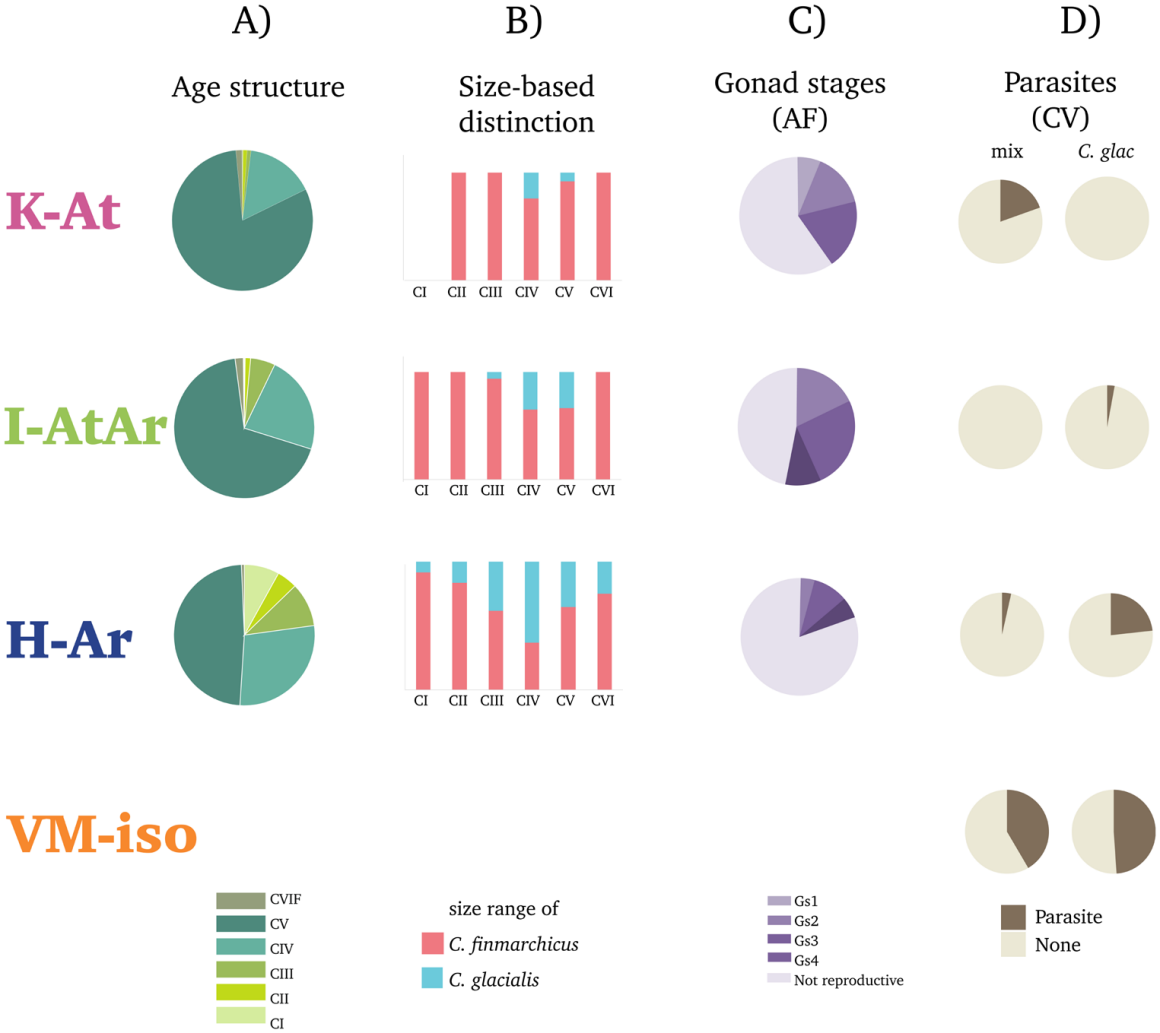
Structural compositions. The results from net samples from formaldehyde‐preserved individuals: (A) *Calanus* age structure—counts of specific life stages in subsamples, (B) size‐based morphological distinction between two species— *C. glacialis* and *C. finmarchicus* across copepodite stages, (C) relative proportions of gonad stages of *Calanus* females; result assessed from photographs: (D) the percentages of *Calanus* CV individuals that hold structures resembling parasites.

In general, the fifth copepodite stage (CV) dominated the population stage structure in each study region (Figure 3A), with the proportion of CV increasing progressively northwards from 48% in H‐Ar through 68% in I‐AtAr and 81% in K‐At. The age structure differed clearly among the studied fjords (G‐test, *p* < 0.001).

The highest abundance of *Calanus* was found in K‐At (1182 ind.m^−3^, 3.5 * 10^5^ ind.m^−2^), followed by 590 ind.m^−3^, 1.3 × 10^5^ ind.m^−2^ in I‐AtAr, and the lowest abundance was found in H‐Ar (226 ind.m^−3^, 0.5 × 10^5^ ind.m^−2^). If we were to distinguish specific life stages only by their size, then the *Calanus* populations would be dominated by *C. finmarchicus* (Figure 3B). The highest overall proportion of individuals within the size range of *C. glacialis* (Kwasniewski et al. 2003; Weydmann and Kwasniewski 2008) was found in H‐Ar, and it was slightly greater in I‐AtAr than in K‐At (Figure 3B). Even though females were not abundant, some were still in a reproductive mode, especially in I‐AtAr and K‐At, with 57% and 40%, respectively (Figure 3C).

The most isolated location, VM‐iso, was characterized by the highest proportion of parasite‐like internal structures in *Calanus* CV (approximately 50% of individuals), regardless of the category (Figure 3D). A high percentage (approximately 25%) of infected individuals was also found in the *C. glacialis* category in H‐Ar and in a mix category in K‐At. Almost none of this phenomenon was observed in I‐AtAr or in *C. glacialis* from K‐At.

### 3.3 Pigmentation

**FIGURE 4:**
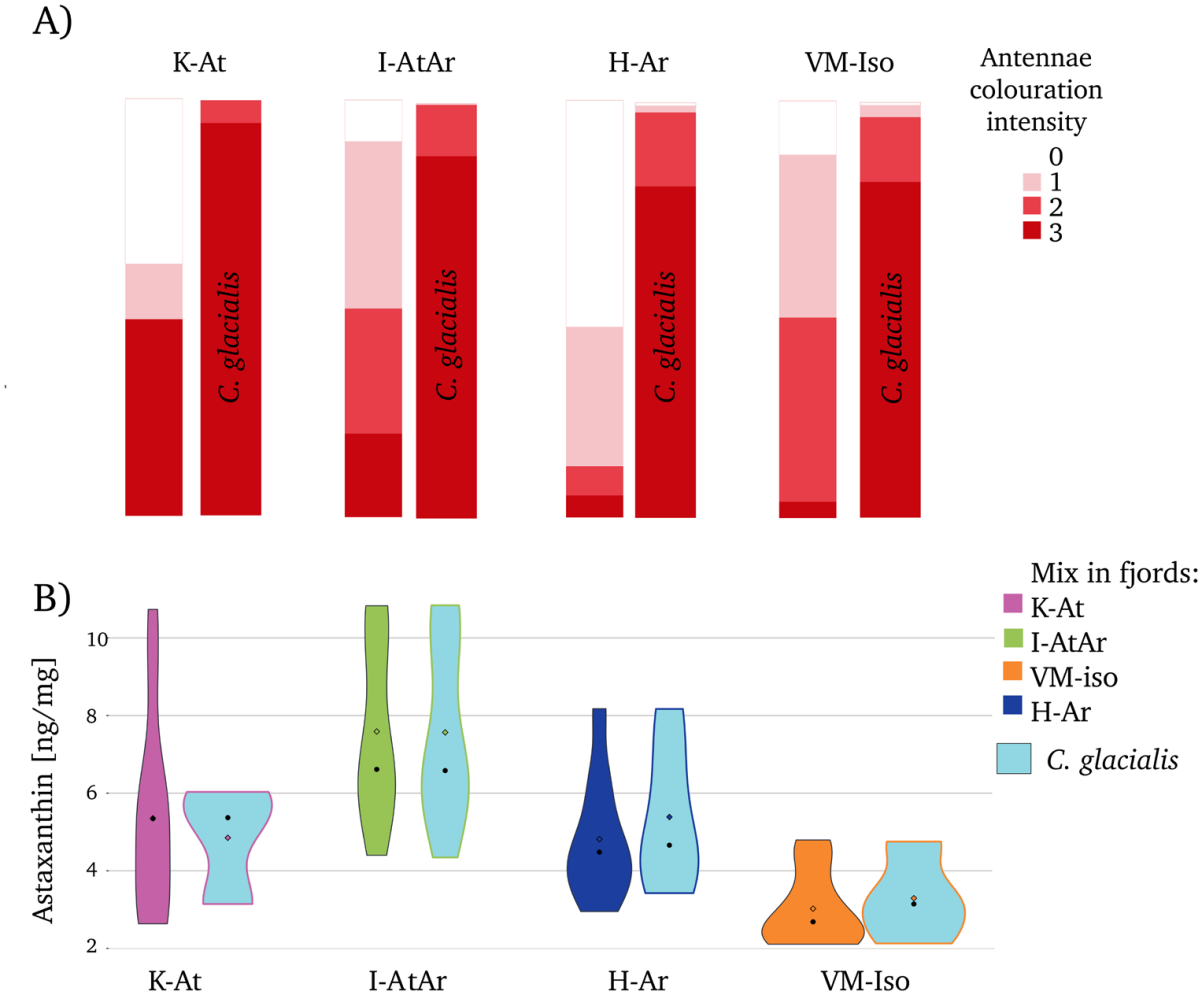
Pigmentation in *Calanus* in specific regions (K‐At: Kongsfjorden, I‐AtAr: Isfjorden, H‐Ar: Hornsund, VM‐iso: Van Mijenfjorden). (A) Image‐based assessment of pigmentation of antennas in mixed (left bars) and normative *C. glacialis* (right bars) individuals; (B) Astaxanthin concentrations derived from HPLC analysis in the mixed category (left, colored violin) and normative *C. glacialis* (right, blue violin) individuals.

Red coloration of antennas was almost always present in normative *C. glacialis* CV individuals (Figure 4A), with slight variation in that the proportion of the intensity of coloration between the most intense (3) to medium (2) levels was greater in the northern fjords (K‐At, I‐AtAr) than in the southern fjords (VM‐iso, H‐Ar). In H‐Ar, the great majority of the mixed group was pale (0) or almost pale (1), whereas in the other three locations, approximately half of these individuals were pale, especially in K‐At (Figure 4A). Despite the large differences in antennae pigmentation between normative *C. glacialis* and the mixture of its smaller individuals with *C. finmarchicus* (Figure 4A) observed in photos of live individuals, no differences between those two groups were observed in terms of astaxanthin pigment concentrations assessed via HPLC (Figure 4B) (Kruskal–Wallis chi‐squared = 6.0031, df = 2, *p* value = 0.05). The only differences in astaxanthin pigments were among the locations (Kruskal–Wallis chi‐squared = 19.199, df = 3, *p* value = 0.00025), with the highest concentrations in I‐AtAr (mix samples were probably dominated by *C. glacialis* ), and intermediate levels in K‐At and H‐Ar, whereas the lowest pigment concentrations were found in VM‐iso (Figure 4B).

### 3.4 Lipid Content

**FIGURE 5:**
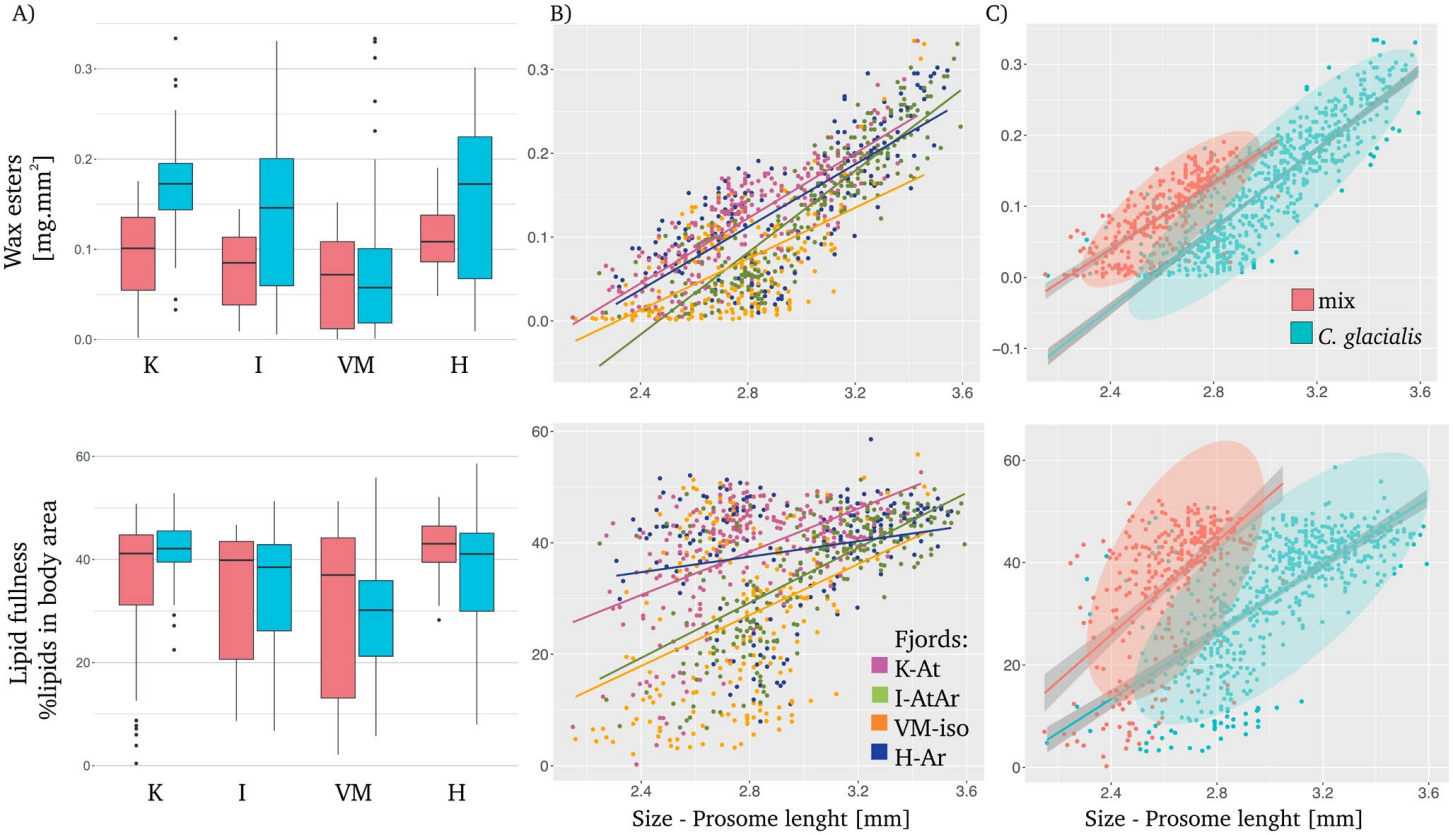
Lipids in *Calanus*. Image‐based estimation of the concentration of wax esters (WE, first row) and their fullness with lipids—the percentage of prosome area covered by a lipid sack (second row). Box plots present the means (lines), quartiles (boxes), the range of most of the data, excluding extreme outliers (whiskers) in the mixed category and normative *C. glacialis* across the studied regions (K‐At: Kongsfjorden; I‐AtAr: Isfjorden; H‐Ar: Hornsund; VM‐iso: Van Mijenfjorden). Scatter plots present the relationship between size and lipids in a region‐oriented and category‐oriented manner. Ellipses are based on multivariate *t*‐distribution, with confidence level 0.95. Linear regression is presented as lines.

Owing to the larger individual body size, the total amount of image‐based estimation of lipids (wax esters) was often greater in the normative *C. glacialis* CV than in the mixed category (Figure 5) (Wilcoxon test, *W* = 68,598 *p* value = 1.423e^−11^). However, there was very high variability in the amount of wax esters within the normative *C. glacialis* group in H‐Ar and I‐ArAt (variance 0.008 and 0.007, respectively), whereas more homogenous results were found in K‐At (*v* = 0.002) (Figure 5). Regionally, the lowest content of wax esters per individual was found in both groups in VM‐iso (Figure 5).

The fullness of the body by lipids (% body area) across size was high, especially in the mix category in I‐AtAr (*v* = 220) and VM‐iso (*v* = 260) (Figure 5). This finding suggests that substantial intergroup differences occur there. Despite this high variability, the overall mean fullness differed between categories (Wilcoxon test *W* = 106,999, *p* value = 0.0007), for example, the values were even greater in a mixture of populations than in normative *C. glacialis* (Figure 5).

The positive relationships between body size and both wax esters content (*r* = 0.79, *p* value < 2.2e^−16^) and fullness (*r* = 0.43, *p* value < 2.2e^−16^) were observed (Figure 5B,C). The increase in lipid fullness with size was steeper in mixed category (*b* = 45.5, *r* = 0.32, *p* = 1.0e^−27^) than in the normative *C. glacialis* (*b* = 32.8, *r* ^2^ = 0.47, *p* = 9.0e^−85^) (Figure 5C).

### 3.5 Diet—Stable Isotopes

**FIGURE 6:**
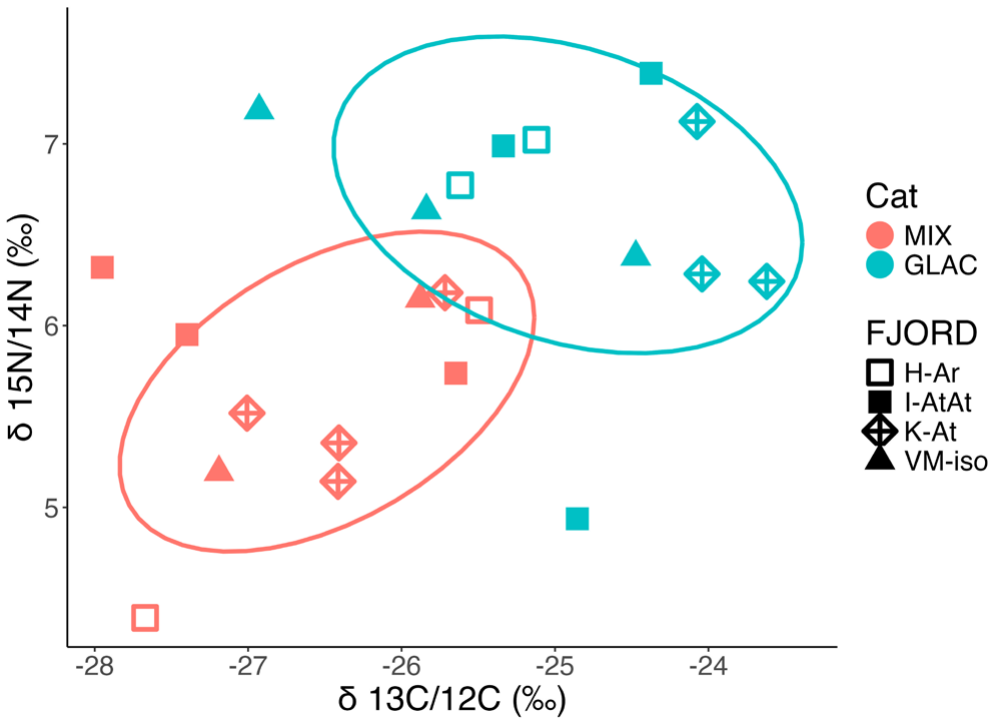
Stable isotopic compositions (δ^15^N and δ^13^C) in *Calanus*, marked by categories (color) of mix and normative *C. glacialis* ; and investigated fjords (shapes, K‐At: Kongsfjorden; I‐AtAr: Isfjorden; H‐Ar: Hornsund; VM‐iso: Van Mijenfjorden). Ellipses are based on multivariate *t*‐distribution, with confidence level 0.75.

The stable isotopic composition did not differentiate fjords, as the wide range of δN and δC values was recorded in each fjord (Figure 6). Typically, normative *C. glacialis* CV individuals had high values of δN and δC, with the smallest intergroup variability found in K‐At. Samples with mix category had mostly lower δN and δC values (Figure 6).

### 3.6 Machine Learning

The performance of the machine learning models was first assessed directly, looking at the segmentation performance. For Model 1, the mean Intersection over Union (IoU) for lipid sac segmentation was 86%, and for prosome segmentation, it was 94.4% (Figure S2). Notably, 95% of the segmentations achieved an IoU value above the 90% threshold. The F1‐score was 0.94 for the lipid sacs and 0.97 for the prosomes. Furthermore, 92% of the prosome segmentations yielded F1‐scores above 0.95, with 65% of these falling within the 0.97–1.00 range. For Model 2, the performance was qualitatively similar.

**FIGURE 7:**
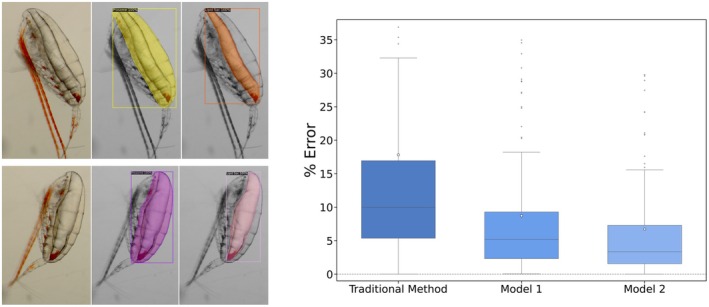
Exemplary images: Two sets of triples in a row: Raw image, image with prosome identified, and image with lipid sac identified for Model 2. Percentages of errors of the LP ratio prediction across manual and machine learning methods on the test set (171 images) adapted to *Calanus* images. The ground truth in each case is based on a detailed manual annotation, while the traditional method is based on a quick manual fit of ellipses performed independently.

Benchmarking via the Mean % Difference between the predicted and ground‐truth LP ratios on the 171‐image test set revealed that the traditional method deviated by 18 percentage points (Figure 7). Model 1 reduced the error to a 9‐percentage point difference. PointRend‐enhanced Model 2 obtained the best score, with a 7‐percentage point difference. This means that Model 2, compared with the traditional method, improved the LP ratio estimation, reducing its error by over 62%.

The automated, ML‐based approach was significantly faster than the traditional approach. While manually outlining the prosome and lipid sack takes several minutes and requires a high level of concentration, large screen, and good manual skills, the performances of both Model 1 and Model 2 showed an average processing time of 1.5 s per (2560 × 1920) image, which included image loading, prosome segmentation, lipid sac segmentation, and ratio computation. Model 1 is marginally faster than Model 2 is, with a difference of < 0.1 s per image.

### 3.7 Traits Overview

**FIGURE 8:**
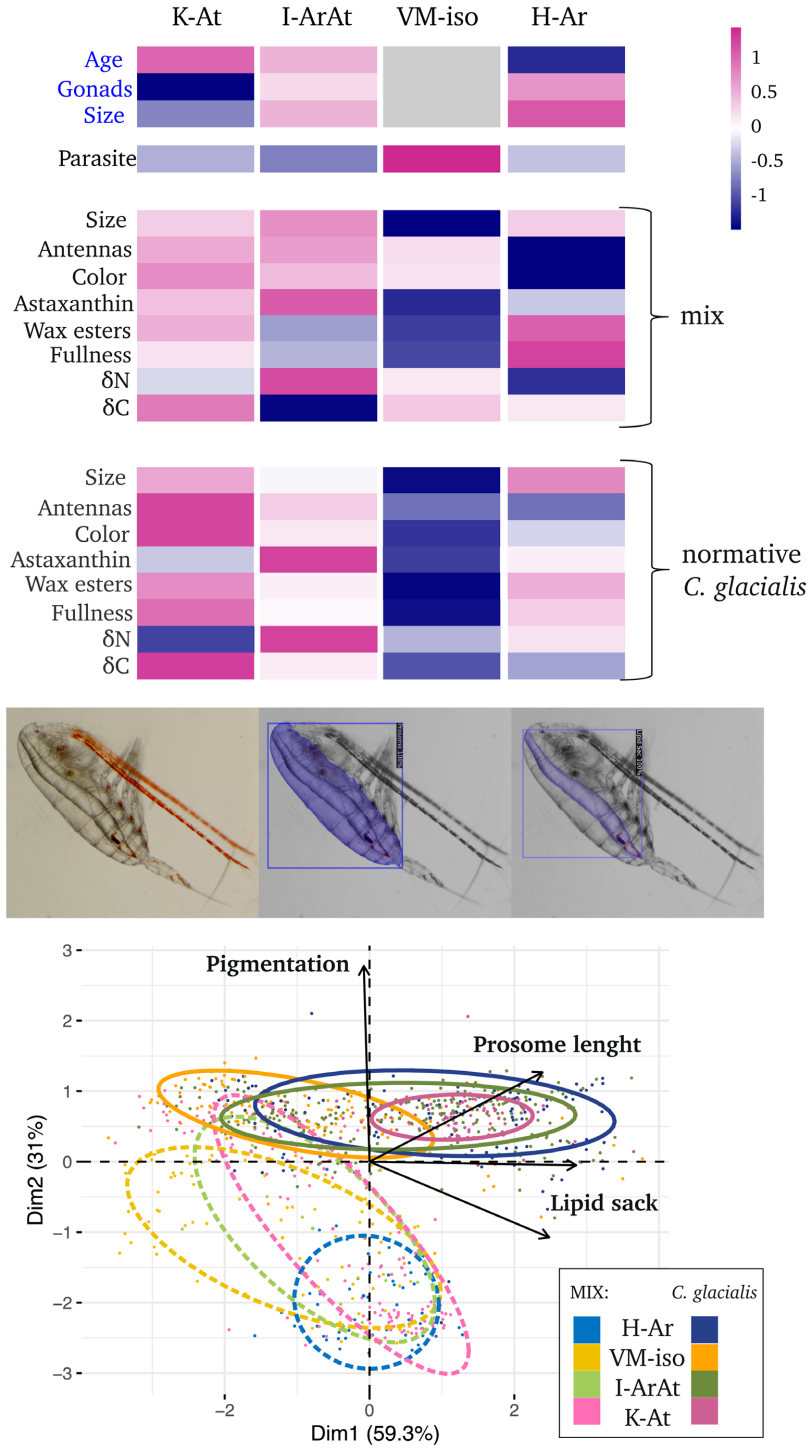
Heatmap and PCA illustrating variability in life history traits of two *Calanus* categories: Mix and normative *C. glacialis* across four fjords (K‐At: Kongsfjorden; I‐AtAr: Isfjorden; H‐Ar: Hornsund; VM‐iso: Van Mijenfjorden). The PCA is based on morphological traits derived from image analysis of live individuals, including prosome length, pigmentation intensity, and lipid sac area as exemplified in images above. Each dot represents a single individual; ellipses indicate 75% confidence intervals. Colors correspond to fjords, with solid lines for the *C. glacialis* category and dashed lines for the mixed group.

When considered collectively, all investigated life‐history traits revealed clear differences in *Calanus* communities across the studied fjords, despite their geographical proximity (Figure 8). The most distinct site was VM‐iso, where individuals exhibited the lowest trait values in the normative *C. glacialis* group. This site was also characterized by a high proportion of parasite‐infected individuals and, in the mixed group, small body size, reduced lipid sac area and fullness, and low astaxanthin concentrations. In contrast, individuals from the northernmost K‐At site were distinguished by generally high trait values, particularly in the normative *C. glacialis* group. Whereas the I‐AtAr was the region with pronounced signals in most traits in the mixed category. The I‐AtAr site displayed strong trait expression in the mixed group, including the highest δ^15^N and astaxanthin concentrations among all locations, regardless of species category. In H‐Ar, the southernmost fjord, individuals from the mixed group showed high concentrations of wax esters and elevated lipid fullness, but notably weak pigmentation (Figure 8). Multivariate analysis of traits accessible via image‐based methods revealed that 59.3% of the total variance was explained by the first axis, which was strongly correlated with prosome length and lipid sac metrics. The second axis, accounting for 31% of the variance, was primarily associated with pigmentation intensity. Morphological trait variability was lowest within the *C. glacialis* group at K‐At and the mixed group at H‐Ar, whereas individuals from VM‐iso exhibited the greatest dissimilarity, with smaller overlap with other fjords.

## 4 Discussion

Our results confirm that the life‐history traits of *Calanus* copepods vary across different Arctic fjords (Figure 8), supporting the hypothesis that hydrographic context shapes trait expression. While seasonal, horizontal, and vertical variability in *Calanus* distribution is well documented (Kwasniewski et al. 2003; Weydmann and Kwasniewski 2008; Vogedes et al. 2014; Trudnowska et al. 2015), our analyses were based on single‐location snapshots focused on individual‐level variability during summer, and thus represent a spatially and temporally constrained comparison across fjords.

Despite this, the observed degree of individual variability—both within species categories and across locations suggests a substantial overlap in trait expression between *C. glacialis* and *C. finmarchicus*. This indicates the presence of an ecological continuum, challenging the long‐standing view of clear ecological and morphological separation between these species in the west Svalbard region. The extensive trait variability observed within each fjord likely reflects the coexistence of multiple cohorts, comprising both local and advected individuals from different generations. These groups may exhibit distinct traits depending on the origin and timing of recruitment.

Rather than providing rigid, species‐specific trait delineations, our findings highlight the complexity introduced by overlapping generations and cohort structures. This intra‐ and interspecific plasticity emphasizes the need for a robust trait‐based approach supported by molecular tools to better understand species functioning in a rapidly changing Arctic environment.

### 4.1 Complex Size Distribution—Different Populations or Generations?

Among the studied copepods, 34% of individuals assigned as *C. glacialis* were smaller (minimum size: 2.2 mm in VM‐iso, 2.3 mm in I‐ArAt & H‐Ar, 2.5 mm in K‐At) than the literature‐based size limit of 2.9 mm (Kwasniewski et al. 2003; Weydmann and Kwasniewski 2008). The size reduction of *C. glacialis* in Svalbard waters has been frequently observed (Gabrielsen et al. 2012; Choquet et al. 2018; Renaud et al. 2018; Trudnowska, Balazy, et al. 2020; Balazy et al. 2023). Because this morphological feature is very flexible, and its variability can be high also seasonally (Pepin and Head 2009; Berchenko and Druzhkova 2023), the smaller sized *C. glacialis* could be represented by later generations that were exposed to different temperatures and food accessibility conditions (Pepin and Head 2009; Persson et al. 2012; Feng et al. 2018). *C. finmarchicus* had at least, two modes of size distribution (Figure 2A), which increased the complexity of understanding the composition of the mixed category even more.

### 4.2 Life Stage Structure—Faster Development or Generational “Smearing”?

Understanding the phenotypical plasticity and resilience of each *Calanus* species may be improved by performing life stage‐specific studies under a range of environmental conditions (Kosobokova 1999; Hirche and Kosobokova 2003), as older life stage individuals might be more stenothermal than younger individuals (Pertsova and Kosobokova 2010). The developmental stage structure of *Calanus* was more advanced at the northernmost, more Atlantic‐influenced location, whereas the youngest community was found at the southernmost, more Arctic location. Part of the reason for this difference could be the slight time shift in the sampling of particular fjords (up to 7 days). The average stage structure was more advanced in 2018 (Trudnowska, Balazy, et al. 2020) and 2019 (this study) than in 2015 and 2016 on the corresponding days of the year (Balazy et al. 2019), suggesting faster development over later years. Whether such faster development will be the future standard resulting in match/mismatch shifts in the *Calanus*‐based Arctic food web remains unclear but afflicting (Renaud et al. 2024).

However, such comparisons are challenging since they are dependent on the region, water temperature, and year (Espinasse et al. 2018; Balazy et al. 2019). We assume that the effect of water masses on phenology observed in our study was similar to that reported in Isfjorden (Espinasse et al. 2018), with a more advanced phenological development where more Atlantic water is advected. Local Arctic populations were previously reported to be delayed in development compared with those advected from warmer locations (Hodal et al. 2012; Weydmann et al. 2013; Skjoldal et al. 2021; Weydmann‐Zwolicka et al. 2022). That is why it has been assumed that mainly the late developmental stages are advected as a result of the faster maturation of the source *Calanus* populations from the south (Skjoldal et al. 2021), and because early life stages (CI–III) of *C. finmarchicus* were rather not observed in the historical records from the Fram Strait. However, the first empirical evidence of locally recruited early developmental stages of *C. finmarchicus* (Tarling, Freer, et al. 2022), along with the increasing contribution of young stages during the summers of the last decade (Gluchowska, Dalpadado, et al. 2017; Gluchowska, Trudnowska, et al. 2017; Weydmann et al. 2018; Strand et al. 2020), clearly shows that also the younger life stages are advected to Svalbard during summer (Kwasniewski et al. 2003; Espinasse et al. 2018; Balazy et al. 2019). All these new observations are critical to re‐establish our knowledge about the life strategies of those key zooplankton species.

The constant presence of early copepodite stages over summer suggests the presence of more than one generation of *Calanus* (Skjoldal et al. 2021). However, their continuous reproduction is highly unlikely, as females were not abundant (1% of all copepodite abundance), and most of them did not have gonads developed for reproduction. Only in Isfjorden did females seem to be in a state of active egg laying (42% of found females), where continuous reproduction of *C. finmarchicus* throughout the summer was previously suggested (Espinasse et al. 2018). Additionally, only singular males were found, and only in advective Isfjorden (1.3 ind.m^3^) and Kongsfjorden (1.8 ind.m^3^). Whereas in the Arctic Hornsund fjord, females finished reproduction at that time in midsummer (senescent gonads), analogously as in the previous year (Trudnowska, Balazy, et al. 2020).

The study of the reproductive strategies of both *Calanus* species from Isfjorden suggests that the mixed reproductive strategies most likely enable the two *Calanus* species to successfully cooccur (Espinasse et al. 2018), and adapt individually to the timing of the pulses of primary production (Niehoff et al. 2002; Søreide et al. 2010; Ji et al. 2013; Ejsmond et al. 2018; Hatlebakk et al. 2022). Moreover, with autumn blooms becoming more common (Ardyna et al. 2014), this prolonged food supply might increase the survival of the generations spawned late.

### 4.3 Pigmentation

While red pigmentation can be a helpful morphological trait in *Calanus* species identification (Nielsen et al. 2014; Choquet et al. 2018; Lindeque et al. 2022), it is also very plastic (Trudnowska, Balazy, et al. 2020), as it is highly dependent on the region, the water mass that determines temperature and food quality, ultraviolet radiation stress, lipid metabolism, or predator pressure (Vilgrain et al. 2023). Another complication is that both *Calanus* species exhibit red pigmentation in their original water mass (Trudnowska, Balazy, et al. 2020); thus, the newly advected *C. finmarchicus* in Atlantic waters most likely could still hold red pigmentation. This is particularly evident in the pigmentation of antennas, which was pronounced in the mixed category in advective fjords, such as Kongsfjorden and Isfjorden. The fact that the mixed category in Hornsund had the highest contributions of the pale individuals suggests that *C. finmarchicus* was out of its comfort zone, similarly as a year before (Trudnowska, Balazy, et al. 2020), or that it was distributed deeper, where pigmentation‐based protection against UVR irradiance is not crucial. The fact that the amount of astaxanthin differed between regions suggests that they had different exposure to this pigment component in their diet because specific precursors of those carotenoids are acquired from their food (Sommer et al. 2006; Mojib et al. 2014). Those differences might also mean that they had different exposure to light or predators in different fjords.

### 4.4 Lipids

The ability of the fifth copepodite stage of *Calanus* to accumulate large amounts of lipids is a flagship feature of those copepods, placing them in a central place in the Arctic food webs (Falk‐Petersen et al. 2009) and making them the conveyors of the lipid pump (Tarling, Belcher, et al. 2022). The content of wax esters and lipid fullness estimated in this study was highly variable across categories, but increased linearly with size, confirming that lipid accumulation is more size‐specific than species‐specific (Renaud et al. 2018; Trudnowska, Balazy, et al. 2020). This increase with size was more linear for wax esters content than lipid fullness, and interestingly, it was sharper at mixed category than normative *C. glacialis* , with greater lipid fullness and wax esters content observed for the corresponding size (Figure 5C).

Overall, the fullness with lipids was lower than that in a previous year (Trudnowska, Balazy, et al. 2020), and the areas of lipid sack were smaller than those assessed for *C. finmarchicus* in the Fram Strait in the late summer of 2019 (Tarling, Belcher, et al. 2022). This may be due to several reasons: They might not have fully benefited from the productive season owing to a mismatch with food availability, or they might have faced high competition.

Another reason for the decreased lipid accumulation could be the presence of parasites. The lowest contents of wax esters per individual were found in both groups at the VM‐iso site, where the greatest number of parasitized individuals was observed. The visually assessed photos clearly revealed a reduced area of the lipid sac, the surface of which was largely occupied by a parasite‐like structure. Gut parasites that have been observed in the form of *Blastodinium* dinoflagellate induce starvation in *Calanus, despite* being cultured at saturated food levels (Fields et al. 2015).

The highest variability in the estimated values of wax esters was observed in normative *C. glacialis* from Hornsund and Isfjorden, implicating that individuals are not necessarily synchronized across co‐occurring populations or generations. Large individual variability in the gut fullness of *Calanus* spp. has been reported previously, even for copepods collected at the same time, location, and depth (Båmstedt 1988; Båmstedt et al. 1992; Pepin and Head 2009).

### 4.5 Diet

Although *Calanus* species are regarded as filter feeders that primarily feed on microscopic algae, it is widely recognized that their diet can be much broader (Søreide et al. 2008; Cleary et al. 2017; Yeh et al. 2020). According to our previous study, both species primarily had an herbivorous, diatom‐based diet in their preferred water masses ( *C. finmarchicus* in Atlantic origin waters and *C. glacialis* in Arctic origin waters). However, their diets were very distinct when they co‐occurred inside the same fjord (Trudnowska, Balazy, et al. 2020). This observation was attributed to the effects of different populations being exposed to various food sources, either due to the co‐occurrence of advected versus local populations or the occupation of different water layers (Trudnowska et al. 2015; Schmid and Fortier 2019). Even though both species occupy very similar niches, they do not necessarily compete for the same resources during recruitment (Hatlebakk et al. 2022). Also, according to the results of this study, there was almost no overlap between the stable composition of normative *C. glacialis* and the mixed category. If *C. glacialis* is more conservative in its diet than *C. finmarchicus* , it might be more vulnerable to environmental changes, especially given that smaller algae, which are expected to become more prevalent, are consumed more efficiently by *C. finmarchicus* than by *C. glacialis* (Hansen et al. 1994; Levinsen et al. 2000).

Considering our results in the frame of general knowledge, zooplankton that feed primarily on phytoplankton in spring typically exhibit high δ13C values, for example, ranging from −22‰ to −19‰ (Tamelander et al. 2006; Søreide et al. 2008). Those that feed on mixed diets, including mixotrophic protists in summer, show lower δ13C values, for example, ranging from −25‰ to −20‰ (Søreide et al. 2008). Additionally, individuals incorporating terrestrial material are depleted in δ13C, with values of approximately −28‰ (Dalsgaard et al. 2003). Based on this, we can conclude that the *Calanus* found in Svalbard fjords during summer have an omnivorous diet, including some terrestrial sources. However, it is challenging to draw conclusions about diet history from an “average field individual” out of a pool of 10 individuals. Therefore, we must be aware that we are working with averages of random individuals whose diets have been highly variable over their lifetime. The δ^15^N values (on average, 6.5 and 5.5 for normative *C. glacialis* and for mixed category, respectively) were lower than those observed in August in other Svalbard locations (Søreide et al. 2008), or in the central Arctic Ocean (Kohlbach et al. 2016), suggesting their lower trophic level in July compared to August. Accordingly, these values are comparable to those recorded for both species in the Hornsund fjordic domain the previous summer (Trudnowska, Balazy, et al. 2020).

### 4.6 Machine Learning for Trait Assessment Automatization

Predicting the population‐wide responses of copepods to climate change is challenging, as such responses are not solely driven by environmental conditions but also are shaped by species‐specific behavioral life‐history traits (Banas et al. 2016). Morphological traits such as body size and the area of a lipid sack are key indicators of *Calanus* life‐history strategies that can now be automatically extracted from images using a convolutional neural network, even from in situ imagery (Maps et al. 2024). By leveraging artificial intelligence, we can now shift our focus from labor‐intensive manual measurements (e.g., contour tracing) toward addressing broader ecological questions, such as the functional roles of *Calanus* in Arctic food webs or the quality of feeding grounds for their predators (Boehnke et al. 2022). This automated approach enables more efficient and scalable assessments of copepod populations, enhancing our understanding of their responses to ongoing environmental change.

## 5 Summary

Advection of water masses with various temperatures, food sources, and populations of *Calanus* is an important process that shapes copepod functioning and, consequently, ecosystem dynamics across the fjords of western Spitsbergen. The pronounced variability and trait overlap observed both within and between *Calanus* categories across fjords highlights their high degree of plasticity and suggests potential adaptability and resilience to ongoing environmental changes. Consequently, the traditionally recognized ecological and morphological distinctions between *C. finmarchicus* and *C. glacialis* are becoming increasingly ambiguous. To better understand the life‐history strategies and biogeographic shifts of these ecologically critical species, future studies should incorporate population‐, generation‐, and cohort‐level analyses supported by trait‐based approaches.

Climate‐driven changes are expected to induce adaptive responses in *Calanus*, likely reflected in: (1) reduced body size, (2) accelerated development, (3) increased interspecific and intergenerational competition, (4) mixed reproductive strategies, (5) altered pigmentation, (6) dietary flexibility in both composition and timing, (7) diverse lipid accumulation strategies affecting their role in the lipid pump, and (8) increased incidence of parasitism.

Crucially, many of these life‐history traits can now be effectively and consistently monitored across spatial and temporal scales using machine learning techniques—offering a reliable, scalable, and innovative tool for tracking ecosystem change in the Arctic.

## Author Contributions

**Emilia Trudnowska:** conceptualization (lead), data curation (lead), formal analysis (lead), funding acquisition (equal), investigation (lead), methodology (equal), project administration (lead), resources (lead), software (equal), supervision (lead), visualization (lead), writing – original draft (lead), writing – review and editing (lead). **Ali Bukhari:** data curation (equal), formal analysis (equal), methodology (equal), software (equal), validation (equal), visualization (equal), writing – original draft (equal), writing – review and editing (equal). **Marta Gluchowska:** conceptualization (equal), funding acquisition (equal), methodology (equal), writing – original draft (equal), writing – review and editing (equal). **Mads Schultz:** data curation (equal), formal analysis (equal), methodology (equal), writing – original draft (equal), writing – review and editing (equal). **Irina Smolina:** data curation (equal), formal analysis (equal), methodology (equal), writing – original draft (equal), writing – review and editing (equal). **Joanna Stoń‐Egiert:** data curation (equal), formal analysis (equal), methodology (equal), writing – original draft (equal), writing – review and editing (equal). **Jędrzej Świeżewski:** data curation (equal), formal analysis (equal), funding acquisition (equal), methodology (equal), software (equal), supervision (supporting), validation (equal), writing – original draft (equal), writing – review and editing (equal). **Kaja Balazy:** conceptualization (supporting), data curation (equal), formal analysis (equal), funding acquisition (equal), supervision (supporting), writing – original draft (equal), writing – review and editing (equal).

## Conflicts of Interest

The authors declare no conflicts of interest.

## Supporting information


Data S1.

## Data Availability

Data are published in the Dryad portal (DOI: 10.5061/dryad.p2ngf1w1w).
