# Supplementary material for: From Pixels to Patterns: Trait Plasticity and Species Overlap of Calanus spp. in Arctic Fjords
Source: Ecol Evol. 2025 May 22;15(5):e71366. doi: 10.1002/ece3.71366 (PMC12098054; doi:10.1002/ece3.71366)
Supplement: Supplementary file 1 — Data S1. [file ECE3-15-e71366-s001.docx]

Suppl. Table 1. Summary of the material collected at each location, how was it used for the 1^st^ and 2^nd^ haul, what kind of measurements, preservation method, analysis and the number of individuals analysed.

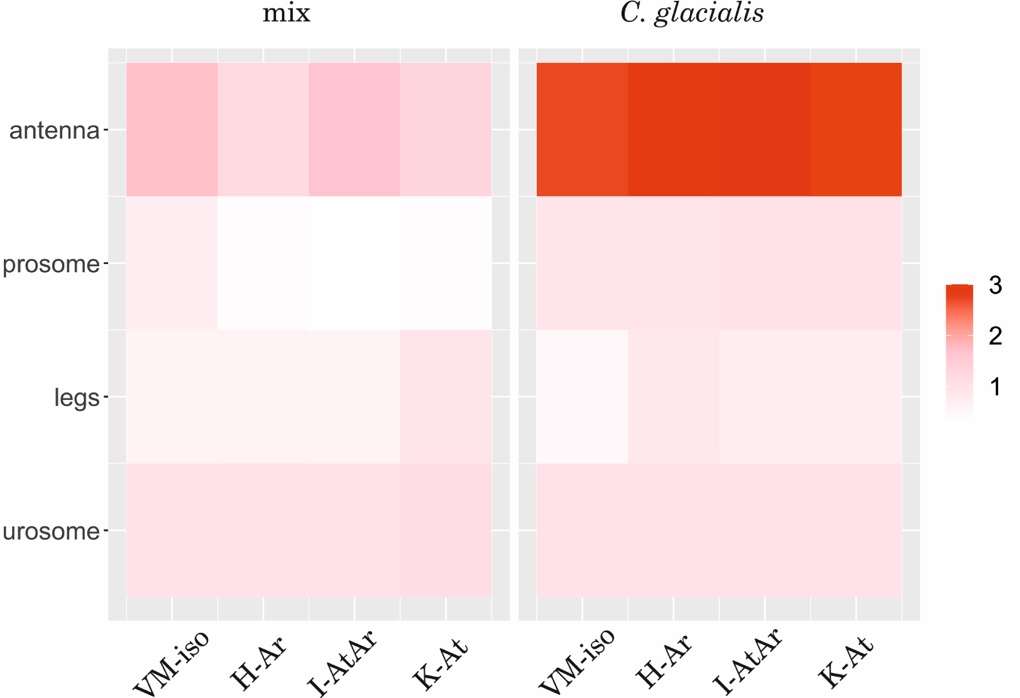


Suppl. Fig 1. Red colouration of genetically identified individuals across specific body parts (rows), averaged over fjords (columns) and categories, with colour signifying the 4 levels of intensity: 3 (>50% red), 2 (10%–50%), 1 (<10%), and 0 (0%, no pigmentation).


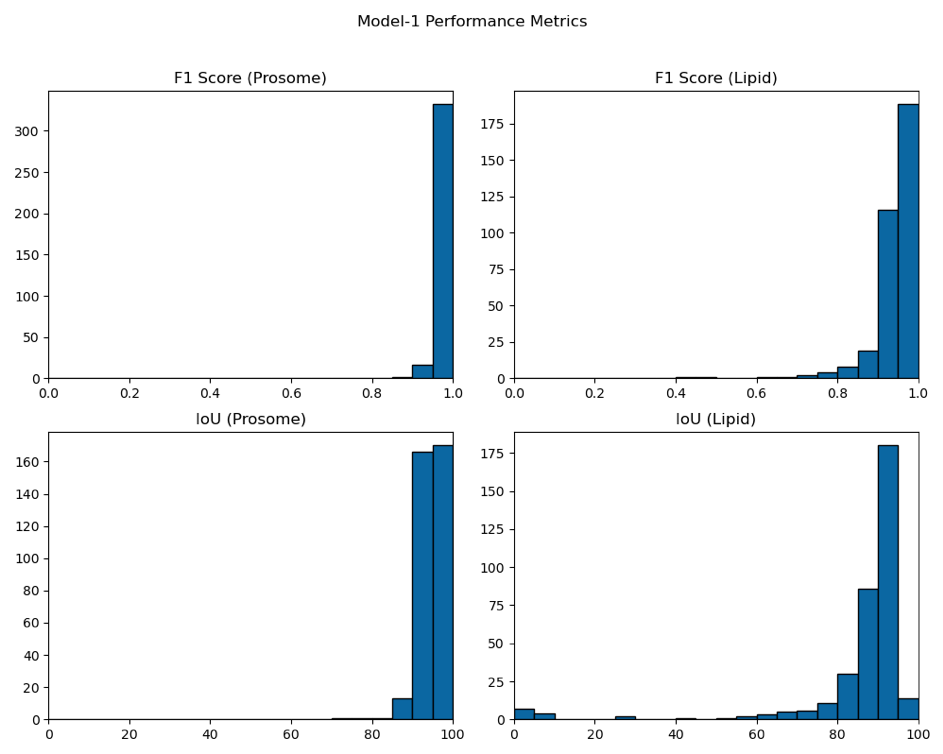


Suppl. Fig. 2. Distribution of IoU and F1-scores on prosome and lipid sac segmentations over the test set (Model 1) of machine learning adapted to images of *Calanus.*
